# Legume intake and risk of prostate cancer: a meta-analysis of prospective cohort studies

**DOI:** 10.18632/oncotarget.16794

**Published:** 2017-04-03

**Authors:** Jie Li, Qi-Qi Mao

**Affiliations:** ^1^ Department of Urology, Lishui Central Hospital, The Fifth Affiliated Hospital, Wenzhou Medical University, Lishui, 323000, Zhejiang, China; ^2^ Department of Urology, First Affiliated Hospital, School of Medicine, Zhejiang University, Hangzhou, 310003, Zhejiang, China

**Keywords:** legume, prostatic neoplasm, meta-analysis, epidemiology

## Abstract

Previous studies regarding the relationship between legume intake and risk of prostate cancer have reported inconsistent results. We conducted a meta-analysis of prospective cohort studies to summarize evidence on this association. A systematic literature search of articles published through June 2016 was performed using PubMed and Web of Science databases. The combined relative risk (RR) with its 95% confidence interval (CI) for the highest versus the lowest intake of legumes was calculated with a random-effects model. Dose-response meta-analysis was also performed for the studies that provided at least three levels of legume consumption. Ten articles (eight cohorts) reporting 281,034 individuals and 10,234 incident cases were identified. The individuals with high consumption of legumes compared with the reference group experienced a significantly reduced risk for developing prostate cancer (RR: 0.85 [95% CI 0.75−0.96], *P* = 0.010). Moderate heterogeneity of RRs was observed across these studies (*P* = 0.064 for heterogeneity, *I^2^* = 45.8 %). Dose-response meta-analysis indicated that the risk of prostate cancer reduced by 3.7% (95% CI 1.5%−5.8%) for each 20 grams per day increment of legume intake. In conclusion, the results from this meta-analysis suggest that a high intake of legumes is associated with a low incidence of prostate cancer.

## INTRODUCTION

Prostate cancer is the second most common cancer in men worldwide, with 1,111,700 newly diagnosed cases and 307,500 deaths estimated to have occurred in 2012 [1]. Age, race/ethnicity, and family history of prostate cancer are the most established risk factors for prostate cancer [2]. Several modifiable factors, such as physical activity [3] and intake of specified vegetables (e.g., carrots [4] and cruciferous vegetables [5]), also have been reported to be associated with prostate cancer incidence, although controversies still persist.

Legumes contain a broad class of bioactive compounds for which anti-carcinogenic roles have been indicated by experimental studies, either directly or through further metabolizing by gut microbiota [6]. In epidemiological studies, legume intake has been linked with a reduced risk of colorectal adenoma [7] and colorectal cancer [8]. Several prospective cohort studies also have investigated whether consumption of legumes is a potential protective factor for prostate cancer with inconsistent results. Diallo et al. [9] and Schuurman et al. [10] reported an inverse association between risk of prostate cancer and legume intake. In contrast, Kirsh et al. [11] found no significant risk reduction associated with consumption of legumes. Hence, this association warrants further investigation.

With the aim of exploring the risk of prostate cancer at different levels of legume consumption, we performed a meta-analysis of prospective cohort studies published up to June 2016.

## RESULTS

### Study selection

**Figure 1:**
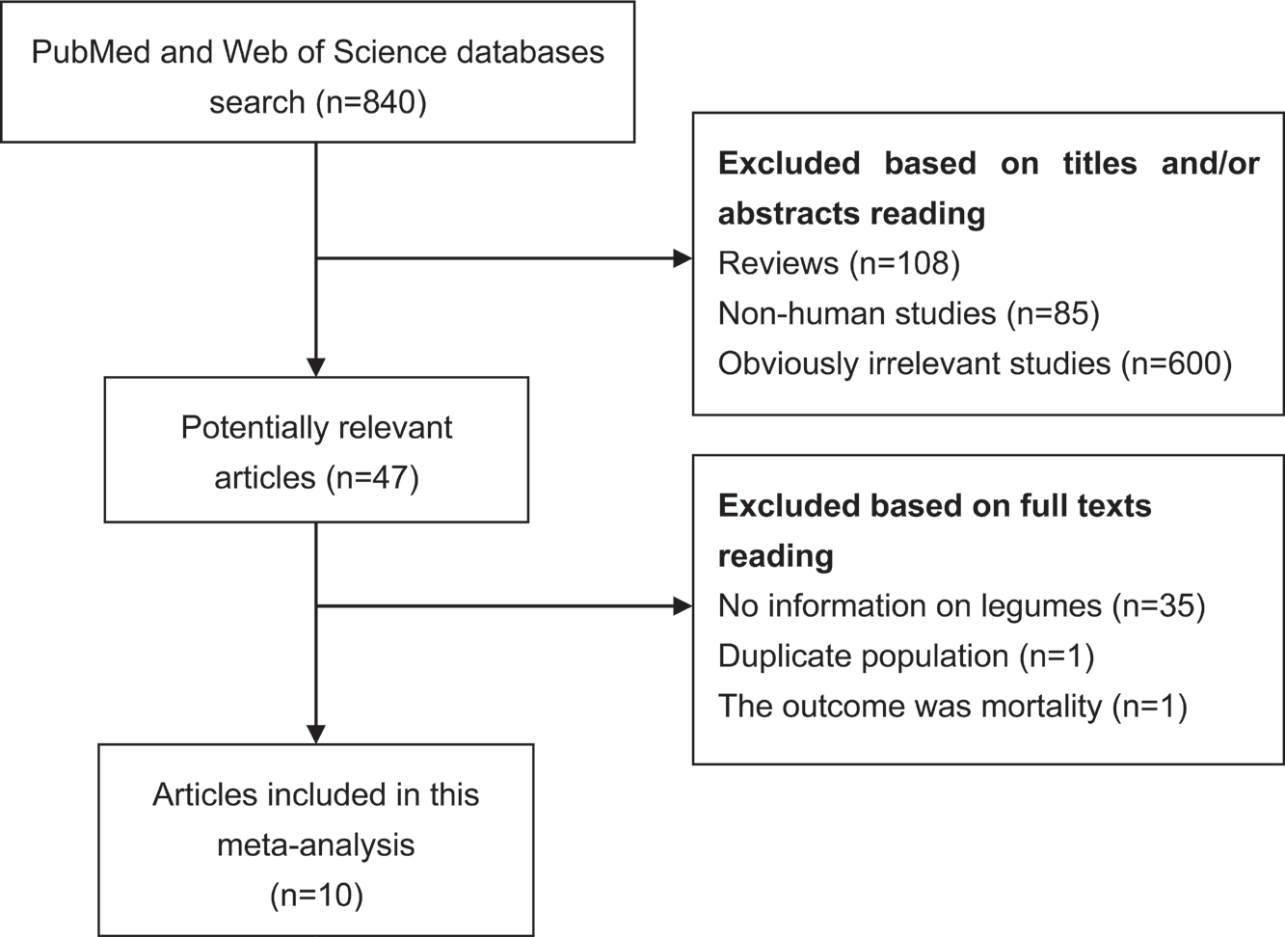
Process of study selection

With the search strategy, 840 unique publications were initially retrieved. After reading the titles and abstracts, 47 articles were considered of interest and full text was retrieved for further evaluation. Thirty-seven of these 47 articles were subsequently excluded and finally 10 articles [9–18] were included in the current meta-analysis (Figure 1).

### Study characteristics

Two included articles published by Park et al. (2008 [18] and 2015 [17]) used the same cohort (Multiethnic Cohort Study). In this meta-analysis, the later study [17] was included in overall analysis as it had a longer follow-up, while the earlier study [18] was adopted in dose-response analysis and subgroup analysis as it provided person-years in each exposure category and RRs for various types of legumes. Similarly, a study performed by Jacobsen et al. [13] was only included in subgroup analysis as it had duplicate population with Mills et al. [15] and only reported one type of legumes. Therefore, a total of eight cohorts reporting 281,034 individuals and 10,234 incident cases were identified. Four cohorts were based in North America, two in Europe, and two in Asia. No studies were based in Africa, Oceania, or South America. Articles were published between August 1989 and March 2016. All of ten articles were prospective cohort studies.

The methodological quality of the included articles was generally good (mean score of NOS = 6.7). All studies had described independent, consecutive sampling of their cohort. Mean or median follow-up duration ranged from 4.2 to 19.4 years. Participants were followed up for an average of over 7 years in the most of articles (70.0%). The sample sizes of the cohorts ranged from 3,313 to 82,483, with the four largest studies recruiting individuals over 40 thousand (Table 1).

**Table 1:** Summary characteristics of the studies included in this meta-analysis

Author	Region	Cohort name	Legume type	No. of Cases	Follow-up (y)	Sample size	Exposure measurement	Outcome ascertainment	NOS	Adjusted variables
Diallo et al., 2016	France	SU.VI.MAX	Whole legumes	139	12.6	3,313	24-h dietary record	Histologically confirmed	8	Age, energy intake, intervention group, number of 24-h dietary records, smoking, education, physical activity, height, BMI, alcohol, family history of prostate cancer, PSA, Ca intake, dairy product intake, and plasma α-tocopherol and Se concentrations
Park et al., 2015	USA	MCS	Whole legumes	7,115	13.9	75,216	FFQ	Cancer registries	7	Age at cohort entry, ethnicity, family history of prostate cancer, education, BMI, height, smoking, history of diabetes, physical activity, alcohol, calcium, lycopene, and selenium
Park et al., 2008	USA	MCS	Whole legumes	4,404	8	82,483	FFQ	Cancer registries	7	Time since cohort entry, ethnicity, family history of prostate cancer, education, BMI, smoking, and energy intake
Kurahashi et al., 2007	Japan	JPHC-BPS	Soy food	307	7.5	43,509	FFQ	Cancer registries	7	Age, area, smoking, drinking frequency, marital status, BMI, and intake of total fatty acids, dairy, vegetables and fruits
Kirsh et al., 2007	USA	PLCO	Dry beans, tofu or soybeans	1,338	4.2	29,361	FFQ	Medical and pathology records	7	Age, total energy, race, study center, family history of prostate cancer, BMI, smoking, physical activity, supplemental vitamin E intake, total fat intake, red meat intake, diabetes, aspirin use, and previous number of prostate cancer screening examinations during the follow-up period
Allen et al., 2004	Japan	LSS	Soy food	196	16.9	18,115	FFQ	Cancer registries	7	Age, calendar period, city of residence, radiation dose, and education
Nomura et al., 2004	USA	J-HCS	Tofu	304	19.4	5,826	FFQ	Cancer registries	7	Age, smoking, alcohol intake, total calories, arm muscle area, and BMI
Schuurman et al., 1998	Netherlands	NCS	Pulses	610	6.3	58,279	FFQ	Histologically confirmed	7	Age, family history of prostate cancer, socioeconomic status, and fruit consumption
Jacobsen et al., 1998	USA	AHS	Soy milk	225	7.6	12,395	Lifestyle questionnaire	Cancer registries	5	Age, BMI, consumption of coffee, whole fat milk, eggs and citrus fruits, and age at first marriage
Mills et al., 1989	USA	AHS	Beans, lentils, peas	180	6	14,000	Lifestyle questionnaire	Cancer registries	5	Age, education, current use of meat, poultry, or fish, current fish only, citrus fruit, dry fruit, index of fruit, nuts, and tomatoes

Abbreviations: No., number; FFQ, food frequency questionnaire; PSA, prostate-specific antigen; NOS, Newcastle-Ottawa Scale; y, year; BMI, body mass index; SU.VI.MAX, Supplémentation en Vitamines et Minéraux Antioxydants; MCS, Multiethnic Cohort Study; JPHC-BPS, Japan Public Health Center-Based Prospective Study; PLCO, Prostate, Lung, Colorectal and Ovarian Cancer Screening Trial; LSS, Life Span Study; J-HCS, Japan-Hawaii Cancer Study; NCS, Netherlands Cohort Study; AHS, Adventist Health Study.

Of all the articles, six investigated the whole legume foods, two studied total soy foods, one investigated tofu, and one focused on soy milk. The ascertainment of prostate cancer based on cancer registries (seven studies), histologically examination (two studies), or medical and pathology records (one studies) (Table 1).

Adjusted RRs could be determined for all cohort studies. Each risk estimate was adjusted for age. Five studies (50.0%) reported an estimate adjusted for at least one of the other two established risk factors for prostate cancer: race (3 cohorts) and family history of prostate cancer (5 cohorts). Four articles provided estimates adjusted for total energy intake. Detailed information on adjustments is present in Table 1.

### Overall analysis

**Figure 2:**
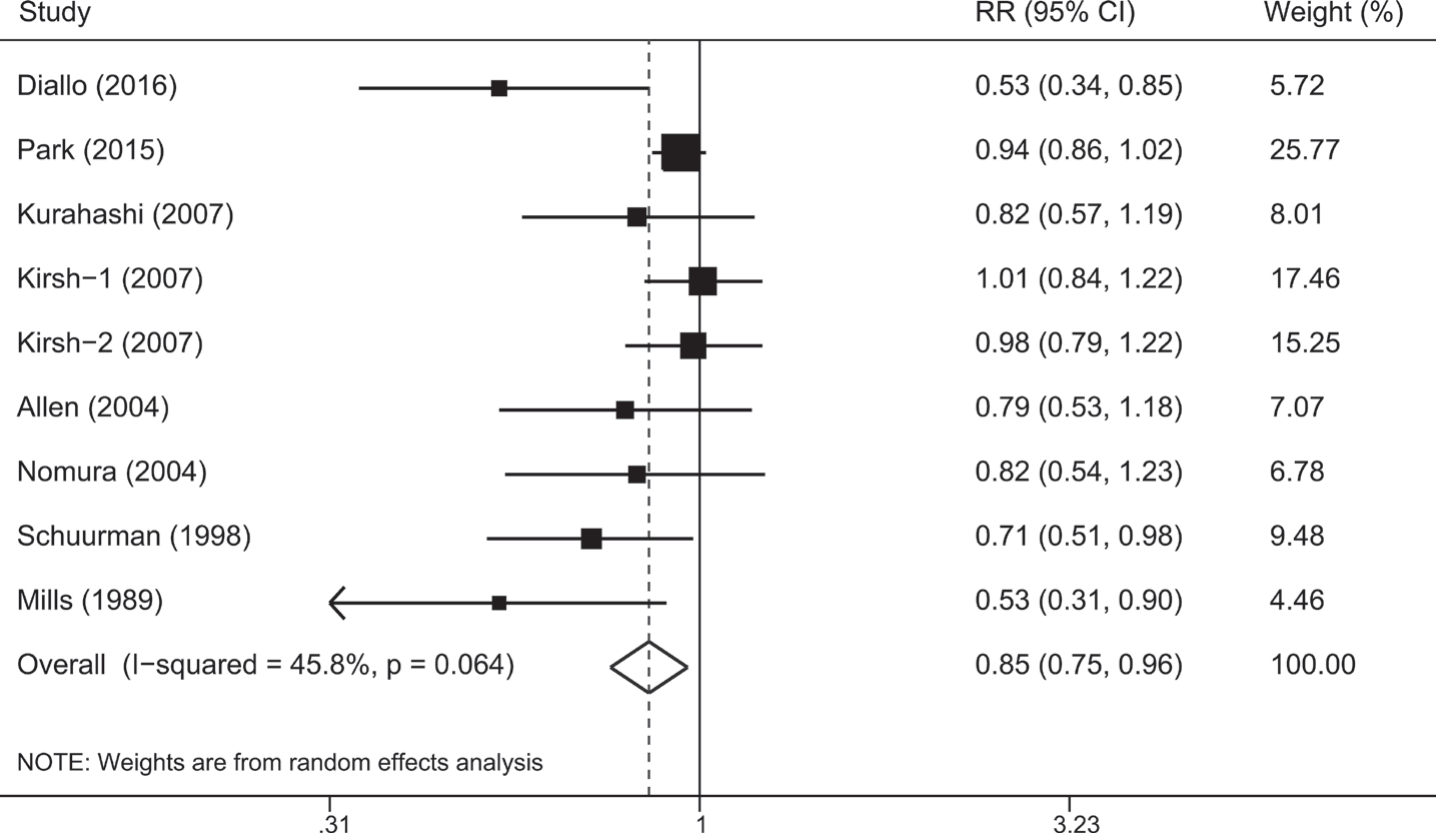
A forest plot showing risk estimates from cohort studies estimating the association between overall legume consumption and risk of prostate cancer

Figure 2 showed the results from the random effects model combining the RRs for prostate cancer. Overall, the individuals with high consumption of legumes compared with the reference group experienced a significantly reduced risk for developing prostate cancer (RR: 0.85 [95% CI 0.75–0.96], *P* = 0.010). Moderate heterogeneity of RRs was observed across these studies (*P* = 0.064 for heterogeneity, *I^2^* = 45.8 %). Therefore, we explored the potential sources (i.e., geographic area, follow-up time, publication year, and sample size) of heterogeneity by using meta-regression. As a result, only the sample size (*P* = 0.033) was identified as a possible source of heterogeneity in the overall meta-analysis. In addition, we also used Galbraith plot to detect studies that might contribute to heterogeneity. As a result, we found that the Diallo et al.’ study [9] was the major source of heterogeneity (Supplementary Figure 1). After excluding this study, there was no significant heterogeneity (*P* = 0.232, *I^2^* = 24.7%), and the combined RR (95% CI) was 0.90 (0.81–0.99).

Considering three studies only reported risk estimates for total soy food or one type of soy food, in order to allow an unbiased comparison, we also computed the RR for whole legume foods from the remaining five studies. Compared with subject with low intake of legumes, the individuals with high consumption of whole legumes experienced a significant reduced risk for developing prostate cancer (RR: 0.85 [95% CI 0.72–0.99], *P* = 0.041). Funnel plot (Supplementary Figure 2), Egger's and Begg's tests all showed evidence of publication bias (Egger, *P* = 0.016; Begg, *P* = 0.022).

### Stratified analyses

Next, we performed stratified analyses based on a number of key study characteristics (Table 2). We firstly carried out stratified analysis according to geographical region, significant protective effect of legumes intake against prostate cancer was observed in European populations (RR 0.64, 95% CI 0.49–0.84, *P* = 0.001), but not in other geographical populations. When stratified by type of legumes, the analysis limited to soy products yielded an RR of 0.89 (95% CI 0.78–1.01, *P* = 0.069). In the subgroup analysis by follow-up time, the RRs (95% CI) were 0.80 (0.64–1.02) and 0.86 (0.71–1.03) for follow-up > 10 years and ≤ 10 years, respectively. Finally, in the stratified analyses by sample size and publication year, statistically significant association was observed in those small studies (RR 0.68, 95 % CI 0.54–0.85, *P* = 0.001) and studies published before 2005 (RR 0.72, 95 % CI 0.59–0.88, *P* = 0.002).

**Table 2:** Stratified analysis of the association between legume intake and risk of prostate cancer

Subgroup	Included studies	Pooled RR (95% CI)	*P*	Heterogeneity		
				*Q*	*I^2^* (%)	*P*
Total	8	0.85 (0.75–0.96)	0.010	14.75	45.8	0.064
Geographical region						
Europe	2	0.64 (0.49–0.84)	0.001	1.04	3.6	0.308
North America	4	0.93 (0.84–1.04)	0.213	5.57	28.2	0.233
Asia	2	0.81 (0.62–1.06)	0.119	0.02	0.0	0.893
Type of legumes						
Whole legumes	5	0.85 (0.72–0.99)	0.041	13.63	63.3	0.018
Soy products	5	0.89 (0.78–1.01)	0.069	5.07	21.1	0.280
Legumes excluding soy products	2	0.93 (0.84–1.03)	0.156	1.09	8.0	0.297
Follow-up time						
> 10 years	4	0.80 (0.64–1.02)	0.067	6.63	54.7	0.085
≤ 10 years	4	0.86 (0.71–1.03)	0.099	8.11	50.7	0.087
Sample size						
> 20,000	4	0.94 (0.87–1.01)	0.075	4.07	1.6	0.397
≤ 20,000	4	0.68 (0.54–0.85)	0.001	3.31	9.4	0.346
Publication year						
> 2005	4	0.92 (0.81–1.04)	0.177	7.24	44.7	0.124
≤ 2005	4	0.72 (0.59–0.88)	0.002	1.86	0.0	0.601

Abbreviations: RR, relative risk; CI, confidence interval.

### Sensitivity analysis and cumulative meta-analysis

**Figure 3:**
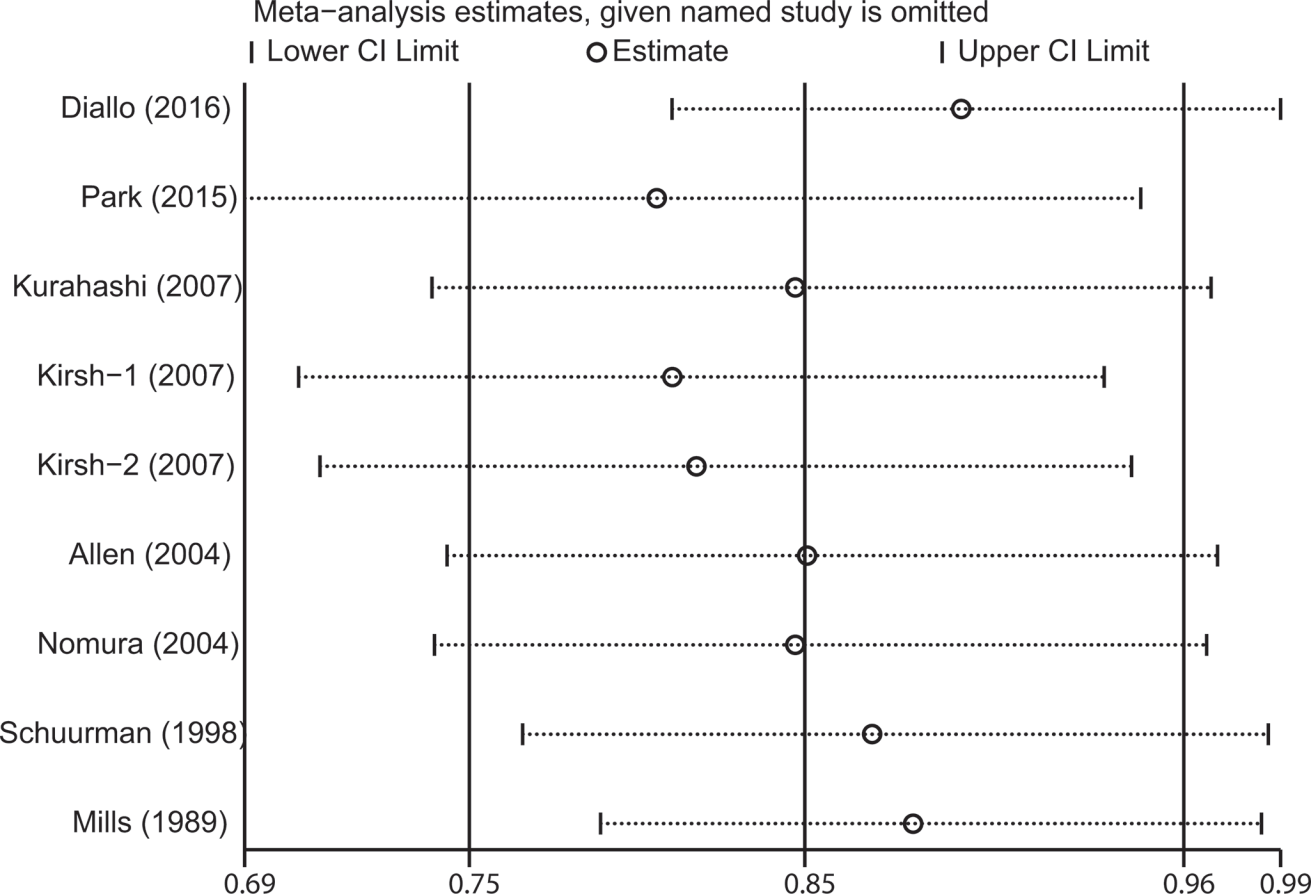
Sensitivity analysis was performed by removing each study in turn and recalculating the pooled relative risk estimates

The influence of each included study on the pooled risk estimate was assessed by repeating the meta-analysis after omitting each study in turn. The results suggested that the combined RR was not dominated by any single study. The RRs ranged from a low of 0.81 (95% CI 0.69–0.95) to a high of 0.90 (95% CI 0.81–0.99) via omission of the study by Park et al. [17] and the study by Diallo et al. [9], respectively (Figure 3).

Cumulative meta-analysis is the process of repeated meta-analysis of individual studies each time adding a new study. In this study, we performed the cumulative meta-analysis according to publication year. As shown in Supplementary Figure 3, The 95% CIs gradually became narrower with increasing sample size, suggesting that the precision of the pooled RRs was progressively boosted by the continual addition of more studies.

### Dose-response relationship and incidence of prostate cancer

**Figure 4:**
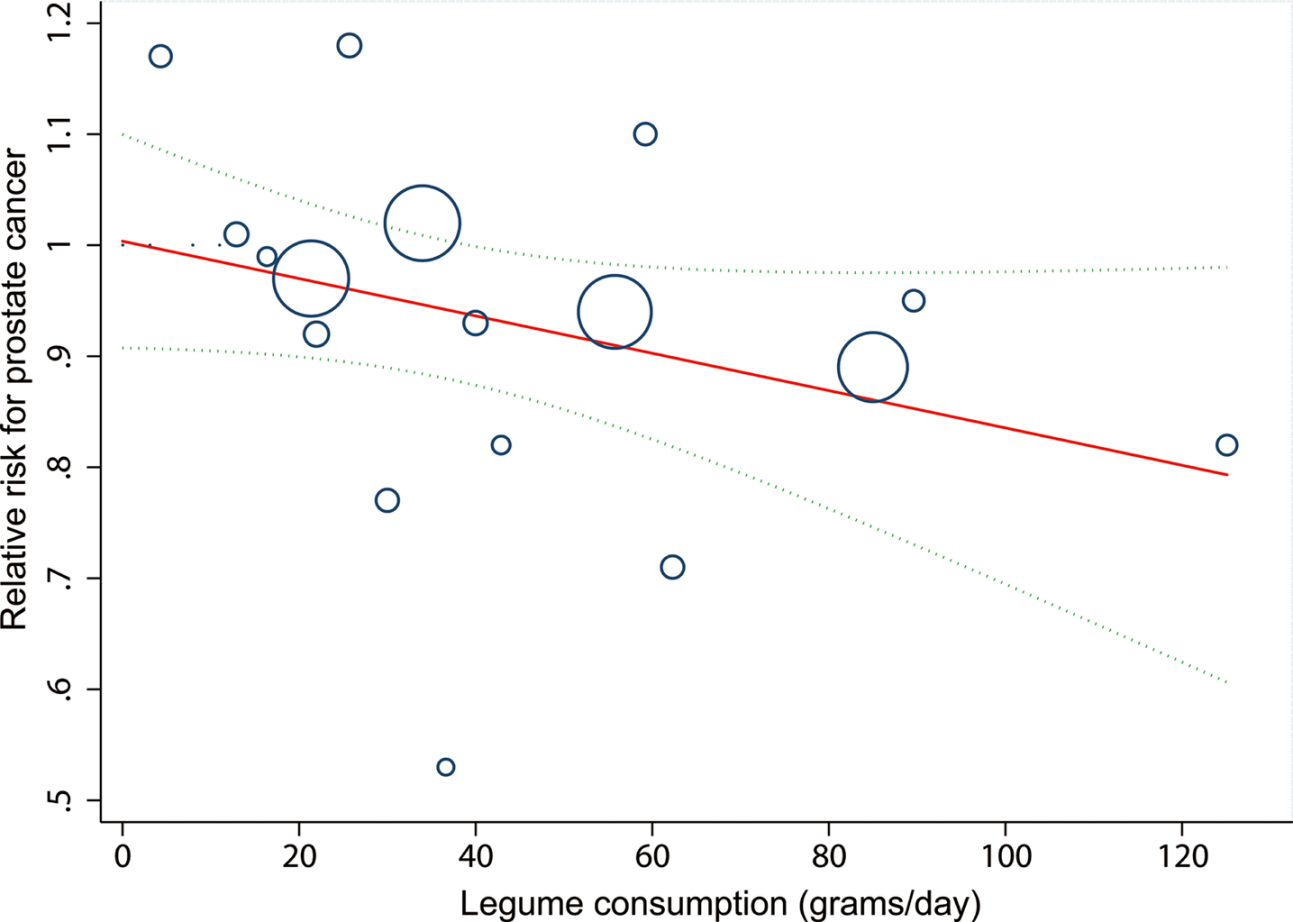
Linear dose-response relationship between relative risk (RR) of prostate cancer and legume consumption Solid line represents the estimated RRs and the dotted lines represent the 95% confidence intervals (CIs). Circles present the dose-specific RRs reported in each study. The area of each circle is proportional to the inverse variance of the RR.

After evaluating dose-response patterns for intake of legumes per day, we observed a linear decrease in prostate cancer risk with increasing legumes consumption (*P* = 0.41 for non-linearity). The risk reduced by 3.7% (95% CI 1.5%–5.8%) for every additional 20 grams per day (for example, an individual who intakes 100 grams legumes per day has a relative reduced risk of 17.2% [95% CI 7.3%–25.8%] for developing prostate cancer compared with someone who never consumes legumes) (Figure 4).

From four population-based studies that reported information on person-years in the highest and lowest categories of legume intake, we could calculate absolute annual rates of prostate cancer cases from the general population: 176.3 cases per 100,000 person-years in the highest group and 152.0 cases in the lowest group.

## DISCUSSION

The current meta-analysis involved approximately 280 thousand participants and 10 thousand patients with prostate cancer from eight prospective cohort studies. To the best of our knowledge, this is the first meta-analysis evaluating the relationship between legume intake and risk of prostate cancer. The results indicated that high level of legume consumption was inversely associated with the incidence of prostate cancer. A dose-response relationship was also observed. The risk of prostate cancer reduced by 3.7% (95% CI 1.5%–5.8%) for each 20 grams per day increment of legume intake.

Two meta-analysis published in 2009 [19, 20] investigated the relationship between soy food consumption and risk of prostate cancer and observed that intake of soy foods was associated with a reduction in risk of prostate cancer. However, these studies included both cohort and case-control studies. When they restricted the analysis to cohort studies in subgroup analysis, the association became weaker. Similarly, in our study we also only observed a borderline association between soy intake and prostate cancer risk in stratified analysis, which was likely due to the small number of eligible studies (*n* = 5) and, hence, insufficient statistical power. However, these results also implied that other types of legumes except soybeans might contribute the protective effects of whole legumes on incidence of prostate cancer, which is worthy of further investigation in future studies.

Heterogeneity is often a concern in a meta-analysis. Moderate heterogeneity was observed among included studies, which could distort the pooled risk estimates. Heterogeneity is caused by variation in definitions and ranges of legume intake, methods of exposure and outcome assessment, and sources of study population. According to the results of meta-regression analysis, we think the difference in sample size is the major source of between-study heterogeneity. In addition, we removed the studies that obviously contributed to the heterogeneity through the Galbraith plot and then repeated the meta-analysis. The relationship still persisted without any significant heterogeneity. These results indicated our findings were robust and less likely affected by the heterogeneity.

A causal relationship between legume intake and prostate cancer risk may be mediated by several mechanisms because of a great variety of anti-carcinogens in legumes. Flavonoids, especially isoflavones, may be the most important contributor to the anti-cancer activity of legumes. Mukhtar et al. reported that dietary flavonoid fisetin could inhibit proliferation, migration, and invasion by binding to β-tubulin and disrupting microtubule dynamics in prostate cancer cells [21]. It also has been demonstrated that isoflavone can inhibit prostate carcinogenesis in the rat [22]. Furthermore, isoflavones sensitize cancer cells to radiotherapy through altered activation of APE1/Ref-1, NF-κB, and HIF-1α [23, 24]. Dietary fiber is another major component of legumes and probably plays a crucial role in this observed association. Dietary fiber may reduce concentrations of circulating androgens through increasing sex hormone-binding globulin concentration [25]. Low circulating levels of testosterone have been indicated to be associated with a reduced risk of prostate cancer [26].

Strengths of our meta-analysis include the strict inclusion criteria, especially restriction to prospective cohort studies, which greatly reduced the likelihood of recall and selection biases. In addition, as individual studies had limited statistical power, our study expanded the sample size and provided more reliable estimates. Finally, the reliability of the findings in sensitivity analyses and Galbraith plot analysis, as well as the significant dose-response relationship, further strengthened our findings.

A potential limitation of this study is the inadequate control of all known confounding factors in included studies, which may bias the results in either direction, toward exaggeration or underestimation of the effect size estimates [27]. Another limitation is the existence of publication bias suggested by Begg's and Egger’ tests. Although we used loose search criteria, gray literature, due to its diverse origins and unpublished nature, may be difficult to find. Small negative studies are also less likely to be published. In addition, like all meta-analyses, our study has the limitation of being a retrospective analysis. Finally, various cut-off points for the categories of legume intake were used in the included studies, which might lead to the heterogeneity and affect the pooled risk estimate.

In conclusion, the results from this meta-analysis suggest that a high intake of legumes is associated with a low incidence of prostate cancer. Considering the limited included studies, further large well-designed cohort studies are warranted to confirm the findings from this meta-analysis. In addition, the mechanisms and active compounds in legumes mediated this relationship remain to be elucidated.

## MATERIALS AND METHODS

### Search strategy

This meta-analysis follows the standards of quality for reporting meta-analyses [28]. We searched the publications recorded in the electronic databases PubMed and Web of Science using the following text and key words in combination both as MeSH terms and text-word form (“legume” or “pulse” or “soy” or “beans” or “lentils” or “peas” or “soybeans” or “tofu” or “soymilk” or “vegetable” or “nutrition” or “diet”) and (“prostatic neoplasms” or “prostatic cancer” or “prostate neoplasms” or “prostate cancer”) and (“prospective” or “cohort” or “nested case-control”). We searched studies published in any language and checked references from these studies to identify other relevant studies.

### Study selection

To reduce varieties between studies, we adopted the following methodological restrictions as the inclusion criteria: studies that provided the minimum information essential to calculate the relative risk (RR) associated with intake of legumes, including cohort and nested case-control studies published as original articles. In instances of duplicate publications, the most comprehensive information with the longest follow-up was used.

### Data abstraction

Articles were reviewed and cross-checked independently by two investigators (J.L. and Q.Q.M). Data on the following characteristics were collected: surname of the first author, publication year, study design, study population, sample size, number of patients who developed prostate cancer, mean or median years of follow-up, study location (defined as Europe, North America, or Asia), type of legumes studied, assessment of exposure, ascertainment of prostate cancer, and reported adjustment for potential confounding factors. When available, we used the effect size estimates from the most fully adjusted model. Any disagreements were resolved by consensus.

### Quality assessment

The quality of each study was evaluated by the same two investigators using the Newcastle-Ottawa Scale (NOS, http://www.ohri.ca/programs/clinical_epidemiology/oxford.asp). This scale is an eight-item instrument designed to assess study population and selection, study comparability, follow-up, and assessment of outcome. One or two points were awarded for each criterion and then points were added up to compare study quality in a quantitative manner. We assigned total points of < 7 and ≥ 7 for low and high quality of studies, respectively.

### Data analysis

Pooled RR and its 95% confidence interval (CI) was calculated by combining the study specific estimates with a random-effects model [29] that accounted for between-study heterogeneity, as significant heterogeneity was anticipated among studies. We calculated the Cochran's Q (the level of significance was set at 0.1) and *I^2^* statistic [30] to evaluate the heterogeneity across studies, applying the following interpretation for *I^2^* < 25%, low heterogeneity; 25%–50%, moderate heterogeneity; > 50%, large or extreme heterogeneity. Various sensitivity analyses, stratified analyses and meta-regression models were performed to explore the potential sources of between-study heterogeneity. In addition to those, the Galbraith plot [31] was used to detect studies that contributed to heterogeneity and re-analysis was performed when the studies possibly leading to the heterogeneity were excluded. Cumulative meta-analysis was also performed through assortment of studies with publication date.

In the dose-response analysis, we included studies that provided at least three levels of legume consumption and person-years in each exposure category. Because most included studies provided categorical data with a range for exposures, we assigned the mid-point in each category to the corresponding RR for each study. When the highest category was open ended, we assumed the width of the interval to be the same as in the preceding category. We used generalized least squares trend (GLST) regression model [32, 33] to assess the pooled dose-response relation between intake of legumes and risk of prostate cancer across studies that had heterogeneous categorizations of legume intake. In addition, we examined a potential nonlinear dose-response relationship by modeling legume intake using restricted cubic splines with three knots at percentiles 25%, 50%, and 75% of the distribution [34]. A *P* value for nonlinearity was calculated by testing the null hypothesis that the coefficient of the second spline is equal to 0.

To calculate the person-years in each exposure category, we extracted data from articles that specified one or more of the following: total person-years of follow-up; sample size and mean (or median) follow-up time; or sample size and cumulative incidence rate. Event rates were expressed as per 100,000 patient-years at risk. Weighted meta-analytic prevalence estimates for outcome were calculated with an inverse-variance random-effects model. The variance was stabilized using the Freeman-Tukey type arcsine square-root transformation [35].

Small study bias (i.e., publication bias) was assessed with funnel plot, Begg's test (rank correlation method) [36] and Egger's test (linear regression method) [37]. All of the statistical analyses were performed with STATA 11.0 (StataCorp, College Station, TX, USA). Statistical tests were two sided and imposed a significance level of *P* < 0.05.

## SUPPLEMENTARY MATERIALS FIGURES


