# Supplementary material for: Legume intake and risk of prostate cancer: a meta-analysis of prospective cohort studies
Source: Oncotarget. 2017 Apr 3;8(27):44776–84. doi: 10.18632/oncotarget.16794 (PMC5546517; doi:10.18632/oncotarget.16794)
Supplement: Supplementary file 1 [file oncotarget-08-44776-s001.pdf]

Legume intake and risk of prostate cancer: a meta-analysis of prospective cohort studies

Supplementary Materials

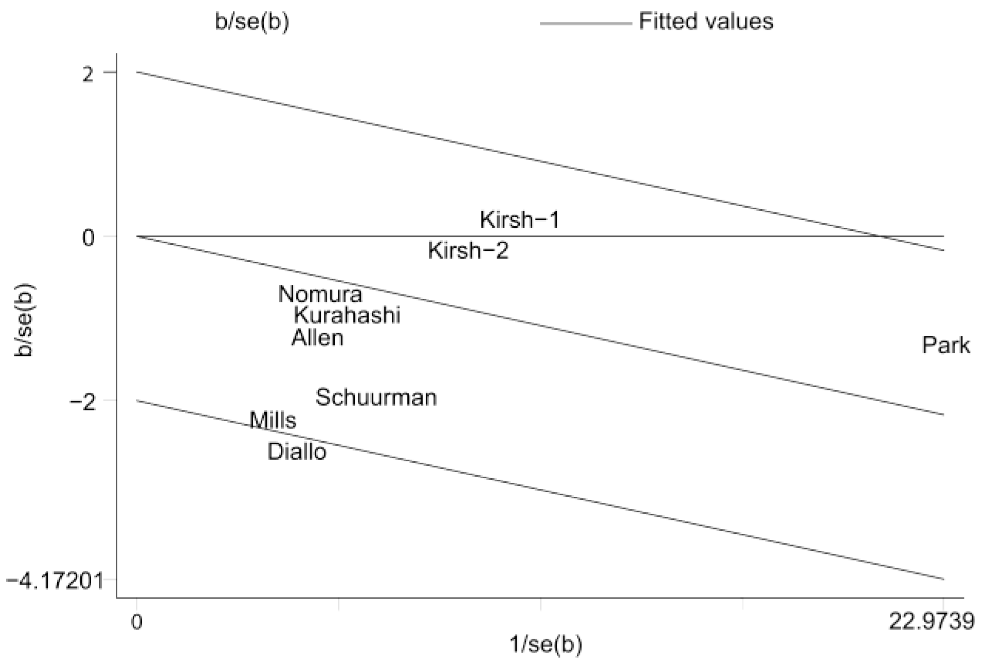

Figure 1: Galbraith plot analysis was performed to evaluate heterogeneity. It indicated that one study was the potential source of heterogeneity.

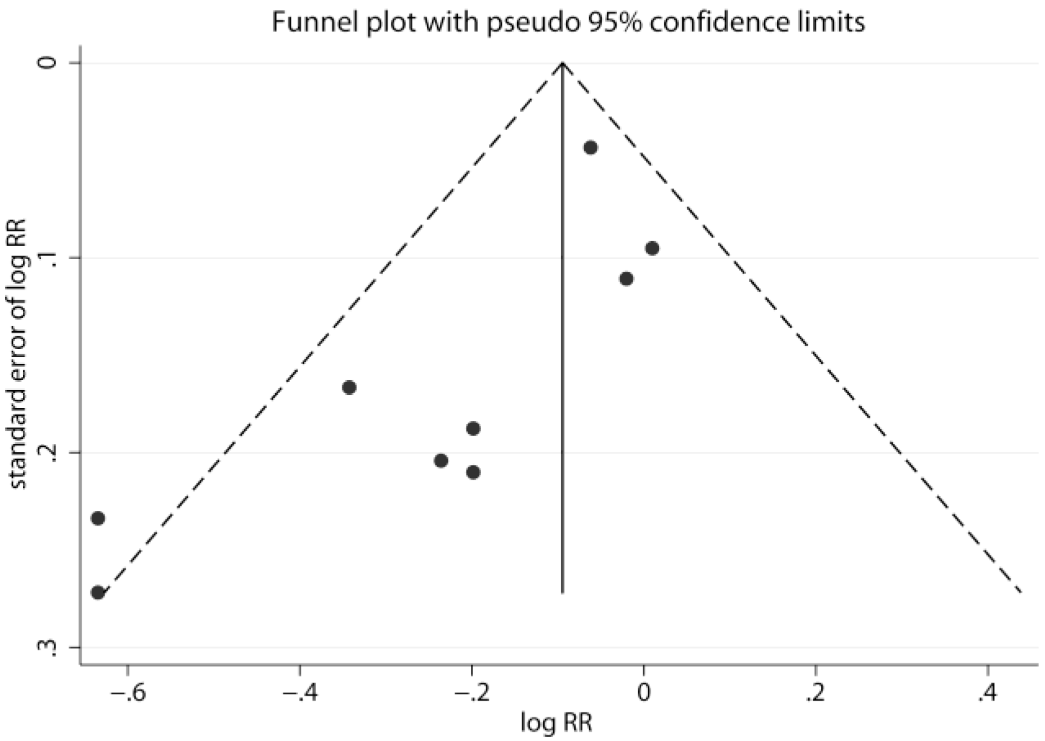

Figure 2: Funnel plot of legume intake and risk of prostate cancer.

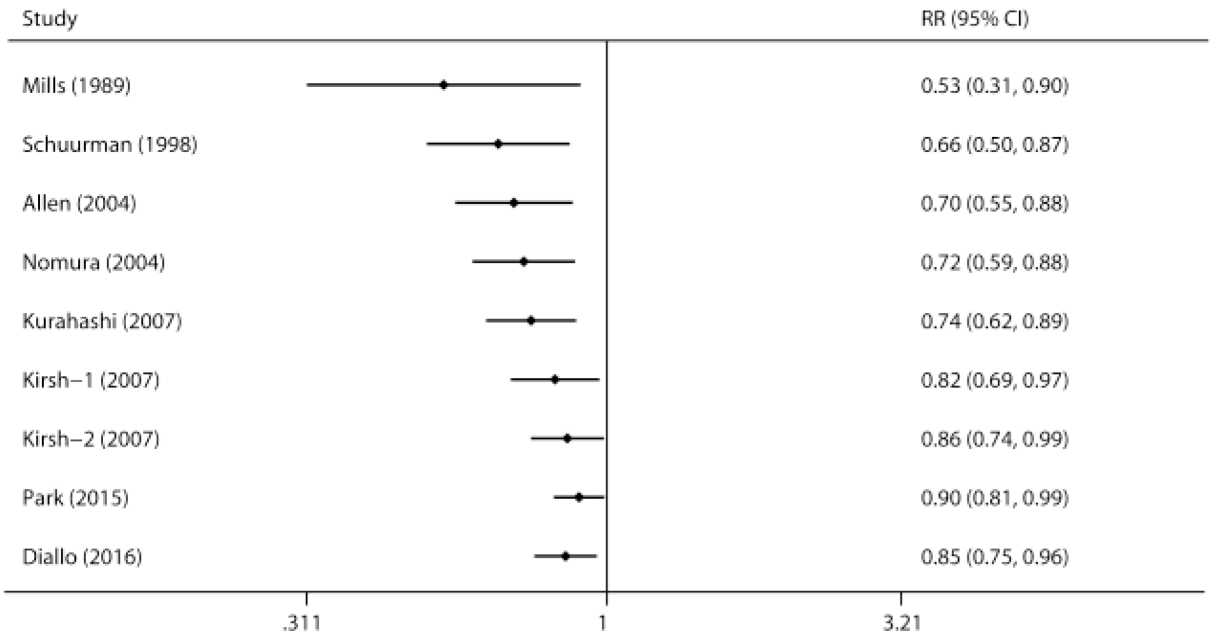

Figure 3: Results from cumulative meta-analysis of the association between legume intake and risk of prostate cancer.
